# Perception of work and health among waste collectors

**DOI:** 10.47626/1679-4435-2022-795

**Published:** 2023-02-13

**Authors:** Daniela Vilas Boas Belarmino, Maria Eduarda Becker Pagani, Andressa Tiemi de Andrade Tanouye, Lucas França Garcia, Ely Mitie Massuda

**Affiliations:** 1 Medicina, UniCesumar, Maringá, PR, Brazil; 2 Consorcio Intermunicipal de Saúde, Associação dos Municípios do Noroeste Paranaense, Paranavaí, PR, Brazil; 3 Programa de Pós-Graduação em Promoção da Saúde, Instituto Cesumar de Ciência, Tecnologia e Inovação, UniCesumar, Maringá, PR, Brazil

**Keywords:** occupational health, solid waste collection, working conditions, saúde do trabalhador, coleta de resíduos sólidos, condições de trabalho

## Abstract

**Introduction:**

Despite the major social and environmental importance of domestic waste collectors, who perform one of the most unsanitary types of work, they still have to deal with the stigma attached to their profession for collecting what society has discarded.

**Objectives:**

To analyze the perception of waste collectors about their work and health.

**Methods:**

Interviews with open-ended questions were conducted with domestic waste collectors from the municipal government staff of a medium-sized city in the state of Paraná, Brazil. A demographic questionnaire was also applied. Answers were analyzed according to Bardin’s content analysis.

**Results:**

Data were collected from 17 participants, all men, with a mean age of 47.7 years. Workers showed different points of view regarding work difficulties and problems, health, people’s perception about their work, and the importance of their work.

**Discussion:**

Although some answers had opposite perspectives, all participants recognized the importance of their work for society, which is not reciprocated. The way collection activities are performed, with collectors using their bodies as an instrument, and the lack of recognition by society may result in physical and psychological problems.

**Conclusions:**

Improving working conditions and making these workers visible to society, considering their indispensability, could promote health strategies directed at this working class.

## Abstract (pt)

**Introdução:**

Os coletores de lixo doméstico, que, apesar da relevância social e ambiental que representam, realizam um trabalho considerado insalubre em grau máximo, ainda lidam com o estigma ligado à profissão por realizarem a coleta do que a sociedade descartou.

**Objetivos:**

Analisar a percepção dos catadores de lixo sobre o seu trabalho e sua saúde.

**Métodos:**

Foram realizadas entrevistas com questões abertas, submetidas à análise de conteúdo de Bardin, com coletores de lixo domiciliar pertencentes ao quadro de funcionários de prefeitura municipal de uma cidade de porte médio do estado do Paraná, além de um questionário social.

**Resultados:**

Foram coletados dados de 17 coletores, todos do sexo masculino, com idade média de 47,7 anos. Diferentes olhares foram apresentados sobre dificuldades, problemas, sua saúde, o olhar da população e a importância de seu trabalho.

**Discussão:**

Por vezes, as falas indicaram opiniões opostas, mas eles reconhecem a importância de seu trabalho para a sociedade, o que não é recíproco. Problemas físicos e mentais podem decorrer da maneira como o trabalho é exercido, usando o corpo como instrumento, e da forma como sentem o reconhecimento de seu trabalho pela sociedade.

**Conclusões:**

Melhorar as condições de trabalho e torná-los visíveis para a sociedade, dada sua imprescindibilidade, são estratégias de promoção da saúde para esses trabalhadores.

## INTRODUCTION

The current standard of living revolves around consumption, which generates waste.^1^ Domestic or residential waste consists of disposable materials generated by home environments, commercial establishments, industries, public places, and service provision.^2^ In Brazil, waste collection is conducted manually by domestic waste collectors.

These workers deal with the stigma attached to their profession for collecting what society has discarded. Despite the great social and environmental relevance of waste collectors, they have been historically marginalized for exercising a profession associated with low educational levels and economic status.^3^ They perform physically and mentally demanding activities during which they are under constant stress due to the chaos of urban traffic^4^ and increased workload. The work process of domestic waste collection uses precarious, mostly manual technology, turning the worker’s body into an instrument for carrying waste.^5^

Waste collection is considered the most unsanitary type of work and exposes workers to many risks, which are aggravated by the physical effort required by the job and may lead to the development of cardiovascular diseases.^6^ In addition, there are also mechanical (injuries due to inadequate disposal of sharps, being run over, falling from the truck, wear and tear of joints), ergonomic (hypertrophy), biological (contact with pathogens), and chemical (lack of care in handling and disposing toxic substances) hazards associated with this type of work.^7,8^

Waste collection is extremely important for society, both in terms of public health and the environment. The objective of this study was to analyze the perception of domestic waste collectors about their work and health.

## METHODS

This was an exploratory, qualitative study of domestic waste collectors from the municipal government staff of a medium-sized city in the state of Paraná, Brazil. A semi-structured questionnaire was used to assess workers’ perception about their health and work. All participants were informed about the objectives and methods of the study and were ensured about the anonymity and confidentially of their answers.

Workers who were absent from work or on leave at the time of data collection were excluded. Workers who agreed to participate in the study signed an informed consent form, according to Brazilian National Health Council Resolution no. 466/12. The research project was approved by the Research Ethics Committee of Universidade Cesumar under no. 3.079.163.

A total of 17 waste collectors were included in the study. The questionnaire was applied between May and July 2019 according to the availability of waste collectors who meet at the Municipal Department of Public Services at the end of the morning shift (between 11:00 and 13:30 pm), at their workplace, according to recommendations by the team coordinator.

Demographic data were collected on age, gender, length of time working as a waste collector, and level of education. The semi-structured, guided interview was based on 4 questions: 2 questions about their perception of work and health, 1 question about their perception of the importance of their work for society, and 1 question about the difficulties and problems they face during work and how people could help to minimize these problems.

The interviews were recorded, transcribed in full, and analyzed according to Bardin’s content analysis^9^ using the mixed methods software QSRNVIVO® for Windows.^7^

## RESULTS

The demographic data of the study sample are described in Chart 1.

**Chart 1:** Demographic data of participants

Waste collector	Age	Length of time working as a collector	Level of education
1	52	11	Incomplete elementary school
2	55	11	Complete elementary school
3	51	24	Incomplete elementary school
4	50	19	Complete high school
5	45	16	Incomplete elementary school
6	42	9	Incomplete high school
7	50	26	Complete high school
8	56	26	Complete high school
9	52	11	Incomplete elementary school
10	50	26	Incomplete elementary school
11	35	5	Complete high school
12	40	9	Incomplete high school
13	51	25	Complete elementary school
14	54	11	Complete elementary school
15	52	26	Incomplete elementary school
16	48	2	Incomplete higher education
17	28	3	Complete elementary school

All participants were men, with a mean age of 47.7 years. Regarding level of education, 29% had incomplete elementary school, 23% had complete elementary school, 11% had incomplete high school, and 23% had complete high school. One participant was enrolled in a university. Mean length of time working as a waste collector was 15.3 years, with the longest being 26 years and the shortest being 2 years.

Participants had different points of view when answering the following question: “How does your job affect your physical health?”. Some participants answered that the job was good for their health:

- Collector 11: [...] It makes us more resistant, improves our immunity. After I started working as a collector, I rarely get sick. I work in the sun, in the rain, I think I got used to it [...]”
- Collector 15: [...] I think it **helps** us to **stay fit**, we are always running [...]”
- Collector 17: [...] It improves our health. I did not work for 2 years and developed **diabetes and high cholesterol**. I came back to work and started running again and everything got better [...]”

However, some collectors said that the job negatively affected their physical health and pointed out problems resulting from physical effort:

- Collector 13: [...] My **feet hurt** from walking too much [...]”
- Collector 9: [...] I have a lot of wear and tear in my shoulders. In fact, I’m undergoing treatment for **bursitis** [...]”
- Collector 2: [...] In relation to **biological risks** (of getting cut by something, for example), it is more dangerous [...]”
- Collector 1: [...] Most of us who have been working this job for a long time have **knee** problems, myself included [...]”
- Collector 3: “[...] sometimes **hitting** the irons hurt [...]”
- Collector 5: [...] Sometimes this job is bad for the **back or legs, arms, head, and mind** [...]”
- Collector 6: [...] We have **tendinitis in the arms** because sometimes we have to use only one arm to perform the service [...]”

The question “How does your job affect your mental health?” also elicited different responses from participants:

- Collector 15: “[...] I think it helps. You **stop worrying** about other things when you are working [...]”
- Collector 8: “[...] I feel **tense** the whole day [...]”
- Collector 3: “[...] Regarding **stress**, it’s hard [...]”

These questions raised interesting points regarding inadequate medical and psychological assistance.

- Collector 6: “[...] We have **tendinitis in the arms** [...] My superiors and the doctor already know, but **nobody does anything**. The doctor gave me a leave of absence when I was doing physical therapy, but I had to come back, and it got worse again [...]”
- Collector 16: “[...] I get **nervous** and feel like I’m being **exploited**. I don’t want to accept this situation, but I have to. If we don’t accept, we end up throwing punches, and we are considered the crazy ones. There are **no psychologists** working here [...]”

The question “What is the contribution of waste collectors to society and how does society perceive this type of work?” elicited the following answers:

- Collector 5: “[...] No one wants do deal with trash. The mayor and the secretary **don’t give a crap about us** [...]”
- Collector 16: “[...] People should be **more careful** when discarding broken glass and such. I’ve cut myself several times, I got 8 stitches on my arm [...]”
- Collector 17: “[...] People think: ‘We are paying you and it does not matter where we throw our trash’ [...]”
- Collector 11: “[...] We often **feel ashamed** when we ask people for water and they don’t give us [...]”
- Collector 15: “[...] Some people **disrespect** us, swear at us, say bad things about us, don’t ask if we’re okay [...]”
- Collector 3: “[...] There is a lot of **prejudice**. Just today, someone threw a bunch of garbage bags full of leaves just to get in our way and delay our work [...]”
- Collector 6: “[...] One of these days a coworker asked to use the bathroom, and the first time they let him, but the second time the guy said the bathroom was occupied, but we saw that he had hid the key and wouldn’t let him use the bathroom anymore [...] ”

The last question referred to “What difficulties and problems waste collectors face during work and how could people help to minimize these problems?”:

- Collector 11: “[...] They should **hire more people and improve the trucks**, which haven’t been fixed for 2 years [...]”
- Collector 16: “[...] There are problems related to **PPE (personal protective equipment), clothes, uniforms, shoes, gloves, and such. They are not adequate for us.** They buy the cheapest ones, and they are not adequate [...]”
- Collector 2: “[...] There are a lot of **structural problems. Trucks and quality PPE are lacking (PPE is available, but the quality is poor)**. We need new trucks, more people working here [...]”
- Collector 15: “[...] It’s difficult when there aren’t enough workers and there is increased workload [...]”

As for how people could help to facilitate their work, separating recyclable and organic waste and correctly handling waste, such as adequately packaging sharp objects, were mentioned.

- Collector 14: “[...] People could **recycle**. They mix everything, doubling our work [...]”
- Collector 15: “[...] They could **recycle** and **properly package things**. Some people even put dirt and broken glass on the bottom of the garbage bag. And when we pick it up and it rips open, it’s our fault. Sometimes we get cut because we don’t know what’s inside the bag. It doesn’t cost anything to separate the trash in different bags [...]”
- Collector 2: “[...] Mostly **separating organic and recyclable waste**, correctly discarding sharps [...]”
- Collector 4: “[...] People who wrongly discard broken glass and we cut ourselves [...]”

## DISCUSSION

Mean participant age (47.7 years) was higher than in other studies: in two medium-sized cities in Southern Brazil, mean age was 26 years^10^; in a city in Minas Gerais, mean age was 33.6 years^11^; and in Dourados, state of Mato Grosso do Sul, mean age ranged from 18 to 31 years.^12^ As a reflection of mean age, mean length of time working as a collector (15.6 years) was also longer than in other studies.^11,12^

Although oldest participants also had the longest time on the job, and younger participants had the least time on the job, this relationship is not linear. This may be explained by the fact that waste collectors are hired through civil service admissions tests, and this type of hiring may happen later in life.

The level of education among participants, of whom more than half had complete or incomplete elementary education, is consistent with data from other studies.^10,11,13^ The worker with the least time on the job (2 years) is 48 years old and is currently enrolled in a university. This shows a trend of overqualification in Brazil, in which overqualified people end up working jobs that were previously occupied by people with lower educational levels, creating a waterfall effect. Less educated people end up occupying jobs that require even less qualification or may even become unemployed.^14^

A number of domestic waste collectors mentioned physical and mental problems resulting from their work. In addition to high exposure to biological risks and work accidents, there were reports of repetitive strain injuries such as pain, tendinitis, and bursitis. Among public cleaning employees in the city of Patos, state of Paraíba, 60% had at least one complaint of pain during working hours, with the lumbar spine being the most affected region.^15^ Collectors of recyclable materials in Curitiba, state of Paraná, who perform similar activities to domestic waste collectors, reported feeling musculoskeletal pain (99.9%), tiredness (95.5%), and headaches (81.8%).^16^ Reports of more than one symptom were common. Domestic waste collectors use their body disproportionately when doing their job due to external aspects such as weather, rhythm, street paving quality, the way people package and discard their garbage, among others, leading to chronic injuries and accidents over time.^17^

The use of PPE should prevent accidents, but workers reported that available PPE is of poor quality and not always used correctly. However, PPE availability does not completely shield workers from exposure to risks, which is why this profession is considered high risk for health issues.^18^ To improve pain and discomfort, participating in workplace exercise before the work shift and getting a massage after the work shift is recommended. These measures, associated with inspection of PPE use and adequate safety measures, could reduce health problems related to waste collection, although completely eliminating them is difficult.^18^

According to Regulatory Standard no. 6,^19^ the employer must provide adequate PPE and demand its use, in addition to training and inspection of correct PPE usage. The employee must correctly use and care for the equipment. The most important PPE for waste collectors are those that protect them from injuries, such as safety gloves, safety boots, breathing masks, safety clothing, and hearing protection, when necessary. However, as reported by study participants, the available PPE is inadequate, “the cheapest”.

The work of domestic waste collectors is considered a complex activity due to the constant and real possibility of accidents, changes in routes or team composition and their implications, unpredictability, instability, uncertainties, among other aspects. Waste collectors are subject to the traffic of people and vehicles, in addition to having to keep up with the team’s and truck’s rhythm, constantly making quick decisions when performing their work.^17^ The time required for learning practical tasks is a summarized indicator of this complexity:

“Beginners stay only on the right side of the running boards for 6 months because if they fall, which is inevitable in the beginning, they are less likely to be run over. Getting on and off the truck without falling alone is an art that takes more than 6 months to master safely. [...] group cohesion, as a collective competence, is needed to reduce the workload, to realize that the garbage bags left by another collector are heavy, and to make the work safer, given that they have to protect themselves while simultaneously observing the traffic, which is an intrinsic risk to the work. There are several responses for the same event, for example, to solve the problem of people who discard their garbage after collection hours.^17”^ (p. 416)

Working as a waste collector requires physical effort because, in addition to collecting garbage bags of different sizes and weights, workers must move between people and vehicles, in streets with varying states of conservation, and under severe climate.^20^ The complexity of the work therefore requires constant management of the worker.

In addition to different physical problems, a significant number of participants reported being subjected to mental stress and often feeling exploited and despised by society. They also mentioned dissatisfaction with medical care and lack of psychological assistance. The main causes of workers’ discomfort are neglect, disrespect, and the lack of recognition, which lead to problems such as mood swings, difficulty concentrating, insomnia, anxiety and feelings of helplessness, frustration, and humiliation.^16,19^

According to Morin,^21^ work plays an important role in people’s life by providing a means through which them insert themselves in the world, exercise their talents, and define themselves, achieving feelings of accomplishment and personal efficiency and giving meaning to their lives. In general, waste collectors are aware of the importance of their work, but this awareness is undermined by the way society judges them. This is evident in the way collectors complain of their working conditions, of how they are treated, and of how society judges them based on their work. People’s disregard for correct waste disposal, leading to accidents with broken glass, needles, knives, and toothpicks, is seen as a devaluation of their work and a disregard for their safety.

The activities performed by waste collectors are painful and often embarrassing, but teamwork helps to deal with difficulties and to find meaning in the work, as seen in aspects such as team distribution and work organization, specific vocabulary, training, tacit knowledge passed on by experienced workers to beginners, dissemination of cultural elements, and the specific way of performing tasks.^22^ Matos et al.^23^ found that, although domestic waste collectors in Fortaleza (Ceará) showed satisfaction with the meaning of their work in the individual and organizational dimensions, there was discrimination and a lack of recognition in the social dimension.

From this perspective, Maslow’s pyramid, or hierarchy of needs,^24^ is worthy of note, in which the conditions to achieve personal and professional satisfaction are established at different levels. The base of the pyramid consists of physiological needs, followed by safety needs on the second level and social needs on the third level. Social needs are associated with feelings of belonging to a social group. At the fourth level, status or esteem needs are associated with self-respect and respect by others. Finally, self-actualization needs occupy the top of the pyramid and are achieved when all other needs are met. According to the psychologist Maslow,^24^ satisfied needs lead to counterproductive behaviors such as fear, frustrations, and anguish.

Therefore, socially invisible workers are those whose occupations generally do not require formal or technical qualifications.^25,26^ Carvalho^27^ emphasizes the precarious working conditions and the social invisibility of this working class, legitimized by the negligence of the State, stating that “the essential is invisible to the eyes” as a parody of the work of Saint-Exupéry, despite the indispensability of waste collection for society.

## CONCLUSIONS

Although domestic waste collectors perceive their work as important for society, they feel professionally undervalued by people who incorrectly discard waste, leading to feelings of disregard for their work and public institutions. Given that neglect, lack of recognition, and lack of medical and psychological assistance were shown to be stress factors affecting the mental health of workers, the mismatch between workers’ perception about the importance of their work for society and the lack of appreciation by the population and the public power is evident. This conflict generates feelings of nonconformity, which are likely expressed through stress.

Divergences on the perception of physical health are possibly associated with the length of time working as a collector, given that using the body as an instrument to perform tasks can lead to or worsen injuries caused by accidents to which workers are exposed to over the years. This study showed that some workers perceive the constant movement and effort required by their work as beneficial, whereas other workers perceive them as detrimental.

This study included workers from the day shift, whose routine is more stable compared with workers from the night shift, who might face different problems in their daily routes. Future studies should include the general population to assess how they perceive waste collectors and the importance of their work for society, which could provide support for public policies promoting health and raise awareness about the real role of domestic waste collectors, providing real meaning to their work.

## References

[1] Hirama ÂM, Silva SS (2010). Coleta seletiva de lixo: uma análise da experiência
do município de Maringá - PR. Rev Tecnol.

[2] Associação Brasileira de Normas
Técnicas (1993). NBR 12980 - Coleta, varrição e acondicionamento de
resíduos sólidos urbanos [Internet].

[3] Barbosa SC, Melo RLP, Medeiros MUF, Vasconcelos TM (2010). Perfil de bem-estar psicológico em profissionais de
limpeza urbana. Rev Psicol Organ Trab.

[4] Velloso MP (1995). Processo de trabalho da coleta de lixo domiciliar na cidade do Rio de
Janeiro: percepção e vivência dos
trabalhadores.

[5] Velloso MP, Valadares JC, Santos EM (1998). A coleta de lixo domiciliar na cidade do Rio de Janeiro: um
estudo de caso baseado na percepção do
trabalhador. Cienc Saude Colet.

[6] Anjos LA, Ferreira JA, Damião JJ (2007). Heart rate and energy expenditure during garbage collection in
Rio de Janeiro, Brazil. Cad Saude Publica.

[7] Velloso MP, Santos EM, Anjos LA (1997). Processo de trabalho e acidentes de trabalho em coletores de lixo
domiciliar na cidade do Rio de Janeiro, Brasil. Cad Saude Publica.

[8] Santos GO (2009). Interfaces do lixo com o trabalho, a saúde e o ambiente -
artigo de revisão. Rev Saude Amb.

[9] Bardin L (2011). Análise de conteúdo.

[10] Cardoso RK, Rombaldi AJ, Silva MC (2013). Nível de atividade física de coletores de lixo de
duas cidades de porte médio do sul do Brasil. Rev Bras Ativ Fis Saude.

[11] Silveira RCP, Silva FM, Ribeiro IKS (2018). Perfil laboral e exposição ocupacional de
cantoneiros de recolha de resíduos sólidos de um
município do Brasil. Rev Enferm Ref.

[12] Lazzari MA, Reis CB (2011). Os coletores de lixo urbano no município de Dourados (MS)
e sua percepção sobre os riscos biológicos em seu
processo de trabalho. Cienc Saude Colet.

[13] Jesus MCP, Santos SMR, Abdalla JGF, Jesus PBR, Alves MJM, Teixeira N (2012). Avaliação da qualidade de vida de catadores de
materiais recicláveis. Rev Eletr Enferm.

[14] Machado AF, Oliveira AMHC, Carvalho NF (2009). Tipologia de qualificação da força de
trabalho: uma proposta a partir da noção de incompatibilidade
entre ocupação e escolaridade. Neco.

[15] Araújo LVPN, Barbosa ALL, Almeida KCS, Vieira TG, Perazzo MT, Bezerra ALD (2016). Prevalence of musculoskeletal symptoms in urban cleaning
agents. Int Arch Med.

[16] Alencar MCB, Cardoso CCO, Antunes MC (2009). Condições de trabalho e sintomas relacionados
à saúde de catadores de materiais recicláveis em
Curitiba. Rev Ter Ocup Univ Sao Paulo (Online).

[17] Vasconcelos RC, Lima FPA, Camarotto JA, Abreu ACMS, Coutinho Filho AOS (2008). Aspectos de complexidade do trabalho de coletores de lixo
domiciliar: a gestão da variabilidade do trabalho na
rua. Gest Prod.

[18] Pedrosa FP, Gomes AA, Mafra AS, Albuquerque EZR, Pelentir MGSA (2010). Segurança do trabalho dos profissionais da coleta de lixo na
cidade de Boa Vista - RR.

[19] Brasil, Ministério da Economia, Secretaria do Trabalho (2018). Norma regulamentadora no. 6 [Internet].

[20] Medeiros IL, Facione GL, Merino EAD, Braviano G (2014). Avaliação de equipamentos de proteção
individual: um estudo sobre os coletores de lixo domiciliar. Des Tecnol.

[21] Morin E (2008). Sens du travail, santé mentale et engagement organisationnel
[Internet].

[22] Oliveira TM, Fontes ARM, Guimarães MRN (2020). A influência da cultura organizacional nos processos de
trabalho dos coletores de lixo domiciliar: um estudo de caso. RGD.

[23] Matos TM, Lima TCB, Paiva LEB, Ferraz SFS (2018). O sentido do trabalho dos garis coletores de resíduos
domiciliares. Rev Gest Organiz.

[24] Maslow AH (2017). A theory of human motivation.

[25] Freiras C (2007). Invisibilidade social dos trabalhadores. Cienc Cult.

[26] Guimarães AZ (1990). Desvendando as máscaras sociais.

[27] Carvalho HM (2018). “O essencial é invisível aos olhos”: a
regulação estatal e coletiva negociada dos direitos
fundamentais imateriais dos trabalhadores coletores de lixo urbano no
Distrito Federal. Rev TST.

